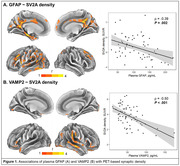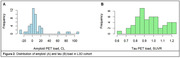# Associations between blood‐based biomarkers, synaptic density and Alzheimer’s disease hallmarks in the brains of nondemented elderly

**DOI:** 10.1002/alz.090738

**Published:** 2025-01-09

**Authors:** Steffi De Meyer, Soha Alali, Maarten Laroy, Thomas Vande Casteele, Margot Van Cauwenberge, Julie Goossens, Charlotte De Rocker, Jeroen Vanbrabant, Eugeen Vanmechelen, Patrick Dupont, Koen Van Laere, Mathieu Vandenbulcke, Louise Emsell, Koen Poesen

**Affiliations:** ^1^ Laboratory for Molecular Neurobiomarker Research (LaMoN), KU Leuven, Leuven Belgium; ^2^ Alzheimer Research Centre, Leuven Brain Institute, KU Leuven, Leuven Belgium; ^3^ Laboratory for Cognitive Neurology, KU Leuven, Leuven Belgium; ^4^ Geriatric Psychiatry, University Psychiatric Center KU Leuven, Leuven Belgium; ^5^ ADx NeuroSciences NV, Ghent Belgium; ^6^ KU Leuven, Leuven Belgium; ^7^ University Hospitals Leuven, Leuven Belgium

## Abstract

**Background:**

Synaptic loss is a critical early pathological hallmark of neurodegeneration, in particular in Alzheimer’s disease (AD), as evidenced by in vitro as well as in vivo PET studies. To date, it is not clear how blood‐based synaptic and AD biomarkers relate to synaptic density in the brains of non‐demented elderly, including those diagnosed with depression.

**Method:**

This cross‐sectional study included 61 older adults with no history of dementia (age [mean±SD] = 71±6 years, MMSE (median[IQR] = 28[3], 64% female, 38% late‐life depression) from the Leuven late‐life depression (L3D) study. Plasma as well as [^11^C]UCB‐J PET (synaptic vesicle protein 2A [SV2A]) were acquired from all participants. Subgroups underwent [^18^F]MK6240 (tau load, n = 51) and [^18^F]flutemetamol (amyloid‐β [Aβ] load, n = 48) PET. Plasma SNAP25, VAMP2 and pTau181 were quantified using Homebrew Simoa assays developed by ADx Neurosciences. Plasma GFAP, Aβ_1‐42_ and Aβ_1‐40_ were quantified using the Quanterix N4PE Simoa kit. Associations between biomarkers and SV2A density were evaluated using voxelwise regression models adjusted for age, sex and depression diagnosis at peak‐level P_uncorrected_ <.05 and cluster‐level P_FWE_ <.05. If significant clusters were detected, Spearman correlations were calculated across the identified clusters for each biomarker. Global brain Aβ and tau load were assessed in Aβ‐ and tau‐vulnerable regions and also correlated with plasma biomarkers.

**Result:**

High GFAP levels associated with low synaptic density within temporal regions (GFAP cluster 1) as well as the precuneus and cingulate gyrus (GFAP cluster 2) with ρ_GFAP_ = ‐0.39 (P =.002, Figure 1A). High VAMP2 levels were associated with lower synaptic density within temporal and frontal and – to a lesser extent – occipital regions (ρ_VAMP2_ = ‐0.55, P <.001, Figure 1B). For the Aβ_1‐42_/Aβ_1‐40_ ratio as well as pTau181 and SNAP25 levels, no associations with synaptic density were observed. Of note, study participants overall demonstrated low global Aβ and tau pathology (Figure 2) and no correlations were found with global Aβ nor tau load for any of the plasma biomarkers.

**Conclusion:**

Plasma levels of GFAP and VAMP2 reflect synaptic density independently of pathological AD hallmarks in nondemented elderly with low evidence of AD pathology.